# Triglyceride Accumulation in Adipocytes Modulated by Insulin Dynamics

**DOI:** 10.3390/ijms262411805

**Published:** 2025-12-06

**Authors:** Tatiana Yu. Plyusnina, Yulia A. Chistyakova, Polina V. Fursova, Sergei S. Khruschev, Diana G. Kiseleva, Alexander M. Markin

**Affiliations:** 1 Department of Biophysics, Biological Faculty, Lomonosov Moscow State University, Leninskie Gory 1-24, Moscow 119234, Russia; 2 Petrovsky National Research Center of Surgery, Abrikosovsky Lane 2, Moscow 119435, Russia; 3 Department of Histology, Petrovsky Medical University, Tsyurupy St. 3, Moscow 117418, Russia

**Keywords:** mathematical modeling, isocaloric feeding, lipolysis, adipocyte triglycerides accumulation, insulin profile

## Abstract

This study examined how meal frequency under isocaloric conditions affects triglyceride accumulation in adipocytes, focusing on the role of insulin dynamics. Using a mathematical model of carbohydrate–lipid metabolism, we simulated feeding regimens from one to eight meals/day while holding calories and macronutrient ratios constant. A simplified model allowed independent variation in insulin peak amplitude, width, and overlap Results show that, relative to thrice-daily feeding (the reference regimen with stable triglyceride content over one month), infrequent meals (1–2/day) reduce, while frequent meals (5–8/day) increase triglyceride accumulation—most strongly in healthy individuals and attenuated in type 2 diabetes, as parameterized from the literature. Crucially, fat accumulation correlates not with average insulin levels but with its dynamic profile. Metabolic flux analysis revealed that triglyceride accumulation is driven not by changes in synthesis rate but by suppression of lipolysis, which depends on the amplitude, duration, and degree of overlap of insulin peaks. Thus, fat mass is shaped not only by caloric intake but by meal timing, which defines the insulin signal’s temporal structure. These findings highlight that insulin dynamics—not mean concentration—govern lipid metabolism, urging dietary guidelines to account for meal pattern, not just composition or total energy.

## 1. Introduction

The number of individuals with overweight, obesity, and type 2 diabetes has been steadily increasing over recent decades, representing one of the major challenges in modern healthcare [1]. These conditions are closely associated with disturbances in carbohydrate and lipid metabolism and increase the risk of cardiovascular diseases, insulin resistance, and metabolic syndrome [2]. Consequently, there is active research aimed at finding effective and safe methods for body weight and metabolic status correction, among which optimizing dietary regimens occupies a central role. However, contradictory scientific data regarding the efficacy of various eating patterns lead to ambiguity in practical dietary recommendations.

A commonly employed strategy remains the reduction in total daily caloric intake. Although this approach is proven effective for short-term weight loss, the results are rarely sustained: most individuals regain their original body weight or even gain additional weight after completing the diet [3]. As an alternative, diets with altered macronutrient ratios but maintained total caloric intake are being studied [4,5,6,7]. Despite certain metabolic effects observed with such regimens, their application may be accompanied by adverse health consequences, particularly in individuals predisposed to metabolic disorders [6,7].

The issue of meal frequency remains particularly controversial. On one hand, proponents of infrequent eating (1–2 times per day) emphasize its effectiveness in reducing body weight, associating this with prolonged fasting intervals and activation of lipolytic processes [8]. On the other hand, frequent small meals (5–6 times per day) are recommended, purportedly to stabilize glucose and insulin level, boost metabolism, and control appetite [9]. However, empirical data on this matter are contradictory, and the mechanisms underlying the impact of feeding patterns on lipid metabolism are still insufficiently understood.

The traditional obesity model, based on the balance between energy intake and expenditure, is gradually being supplemented by alternative concepts, notably the carbohydrate–insulin model [10]. According to this model, a high-carbohydrate diet leads to hypersecretion of insulin, which in turn promotes fat accumulation in adipocytes and inhibits its mobilization. Although this concept currently has limited empirical support and remains controversial [11], it highlights the importance of studying insulin’s role not only as a hormone regulating glucose level but also as a key factor determining the balance between lipogenesis and lipolysis.

Adipocytes, the primary cells of adipose tissue, serve as the main site for energy storage in the form of triglycerides. Insulin plays a central role in regulating triglyceride accumulation: it stimulates the transport of glucose and fatty acids into the cell, activates triglyceride synthesis, and, importantly, suppresses lipolysis [12,13]. Insulin secretion, in turn, depends on the dynamics of glucose entry into the bloodstream, which is determined by the feeding pattern. Despite extensive research on plasma glucose and insulin dynamics [14,15,16], as well as changes in insulin resistance under various diets and pathologies [17], the direct effect of dynamic characteristics of the insulin response, such as amplitude, duration, and frequency of peaks, on triglyceride accumulation in adipocytes remains insufficiently studied. There is limited empirical data directly linking meal frequency to triglyceride accumulation via insulin dynamics under isocaloric conditions.

Mathematical modeling provides a powerful tool for analyzing complex metabolic systems, particularly the carbohydrate–lipid metabolism, allowing the isolation and evaluation of contributions from individual factors that are inaccessible through standard empirical studies. Previously developed models described the dynamics of glucose, insulin, and free fatty acids in plasma [18], the effects of caloric restriction on insulin resistance [19], or the impacts of isocaloric diets with varying macronutrient ratios [20]. However, these models do not consider the influence of the dynamic characteristics of hormonal responses.

In this work, a mathematical model of the carbohydrate–lipid metabolism in plasma and adipocytes is presented, incorporating insulin regulation of key metabolic processes. Using this model, we investigated how meal frequency, under constant daily caloric intake, affects triglyceride accumulation in adipocytes, and proposed that the dynamic insulin profile may serve as a critical factor regulating the balance between lipolysis and fat storage.

## 2. Results

### 2.1. General Scheme of Lipid Metabolism Reactions

In this study, changes in body fat mass were assessed by estimating triglyceride accumulation in adipocytes. This evaluation method is simplified but is considered acceptable at an introductory level as it is widely accepted that adipocyte number remains constant in adults [21]. Therefore, changes in intracellular triglyceride levels directly reflect the corresponding changes in body mass driven by alterations in adipose tissue volume.

**Figure 1:**
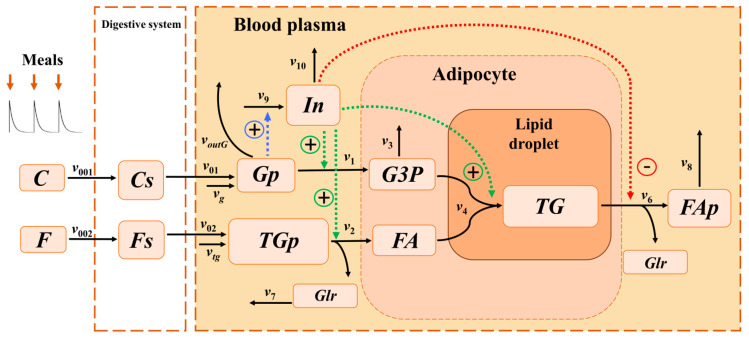
A scheme of the main reactions of triglyceride synthesis. *F*—fats, *C*—carbohydrates ingested with food *Fs*, *Cs*—fats and carbohydrates in the digestive system. Blood plasma metabolites: *TGp*—triglycerides, *Gp*—glucose, *FAp*—fatty acids, *Glr*—glycerol, *In*—insulin. Adipocyte metabolites: *FA*—fatty acids, *G3P*—glycerol-3-phosphate, *TG*—triglycerides of the lipid droplet. *v_i_*—rates of the corresponding reactions. Green dashed line—activation of a reaction by insulin. Red dashed line—inhibition of a reaction by insulin. Blue dashed line—activation of insulin production by glucose.

To investigate the mechanism of triglyceride accumulation at different feeding frequencies, a multi-compartment model was created, integrating processes in the digestive system, blood plasma, and adipocyte. The model included all key reactions involved in triglyceride synthesis (Figure 1).

Based on the approaches presented in [19,22], protein metabolic processes were excluded from the model, as their impact on lipid and carbohydrate metabolism under physiological conditions is insignificant. The intake of food into the digestive system was modeled as a periodic input of macronutrients carbohydrates *C* and fats *F* (see Section 4 for detailed description). This input determined corresponding periodic changes in all metabolites within the model. Carbohydrates *C* entered the digestive system with food at a rate of $v_{001}$, and fats *F* at a rate of $v_{002}$. Carbohydrates *Cs* and fats *Fs* in the digestive system were then transported from the digestive system into the blood plasma as glucose *Gp* and triglycerides *TGp* at rates of $v_{01}$ and $v_{02}$, respectively.

In the blood plasma, triglycerides (*TGp*) underwent lipolysis, yielding one molecule of glycerol (*Glr*) per one molecule of *TGp*, which was transported to other organs and tissues at a rate of $v_{7}$, and three molecules of fatty acids (*FA*), which entered the adipocyte at a rate of $v_{2}$. In addition to the intake of triglycerides *TGp* from food, their influx from the liver was considered at a constant rate $v_{tg}$.

Glucose (*Gp*) was transported from blood plasma into adipocytes, where it underwent glycolysis to form glyceraldehyde-3-phosphate (*G3P*), with an effective reaction rate of $v_{1}$. In addition to dietary glucose uptake, a constant flux of glucose from the liver was accounted for, with rate $v_{g}$. The outflow of glucose (*Gp*) from plasma to other organs was modeled as $v_{outG}$. A fraction of *G3P* molecules was allocated to other metabolic pathways of other cellular metabolic processes at rate $v_{3}$. The remaining *G3P* served as a source of the glycerol backbone and reacted with fatty acids (*FA*) to form triglycerides (*TG*) stored in the lipid droplet of the adipocyte at rate $v_{4}$. One molecule of triglyceride was synthesized from one molecule of *G3P* and three molecules of *FA*.

Triglycerides within the adipocyte lipid droplet (*TG*) underwent lipolysis, yielding one molecule of glycerol (*Glr*) and three molecules of fatty acids (*FAp*) per triglyceride molecule; these fatty acids were subsequently transported back into the bloodstream. Lipolysis of *TG* followed by the export of fatty acids (*FAp*) into plasma was described by a reaction with rate $v_{6}$. Fatty acids in plasma (*FAp*) were then transported to other organs and tissues at rate $v_{8}$.

### 2.2. Mathematical Model of Triglyceride Accumulation in Adipocytes

All reactions, except for triglyceride lipolysis in adipocytes ($v_{6}$), were formulated according to the law of mass action. To describe triglyceride lipolysis ($v_{6}$), it was taken into account that the concentration of triglycerides constituting the lipid droplet within the adipocyte was substantially higher than that of other metabolites. Taking into account this assumption, we assumed that the hydrolysis of lipid droplet triglycerides proceeds under substrate-saturated conditions, i.e., the reaction rate was independent of triglyceride concentration.

The model was described by the system of differential Equation (1) of the form: $\frac{dX_{i}}{dt}=\;\sum v_{i},$ where *X_i_* denoted the concentration of *i*-th metabolite in either blood plasma or in adipocytes, $v_{i}$ represented the net rate of change due to transport, synthesis, or degradation (see Table 1). To convert fluxes from concentration-based units (e.g., mmol L^−1^h^−1^) to absolute amounts (mmol h^−1^), each reaction rate was multiplied by a compartment-specific volumetric scaling factor: linearly for unimolecular reactions and quadratically for bimolecular reactions, reflecting the dependence of reaction flux on molecular stoichiometry and compartment volume. Specifically, $f_{1}$ scaled plasma reactions, and $f_{2}$ scaled those occurring in adipose tissue.

(1) $$\left\{\begin{array}{l} \frac{dC}{dt}=-v_{001}\\ \frac{dF}{dt}=-v_{002}\\ \frac{dCs}{dt}=v_{001}-v_{01}\\ \frac{dFs}{dt}=v_{002}-v_{02}\\ \frac{dGp}{dt}{\cdot f}_{1}=v_{g}+v_{01}-{f_{1}\cdot v}_{1}-v_{outG}\\ \frac{dIn}{dt}{\cdot f}_{1}=v_{9}{\cdot f}_{1}-v_{10}{\cdot f}_{1}\\ \frac{dTGp}{dt}{\cdot f}_{1}=v_{tg}+v_{02}-f_{1}\cdot v_{2}\\ \frac{dGlr}{dt}{\cdot f}_{1}=v_{2}{\cdot f}_{1}-v_{7}{\cdot f}_{1}\\ \frac{dFAp}{dt}{\cdot f}_{1}=3\cdot v_{6}-f_{1}\cdot v_{8}\\ \frac{dG3P}{dt}{\cdot f}_{2}=2\cdot v_{1}\cdot f_{1}-v_{4}{{\cdot f}_{2}}^{2}-{f_{2}\cdot v}_{3}\\ \frac{dFA}{dt}{\cdot f}_{2}=3\cdot v_{2}\cdot f_{1}-3\cdot v_{4}{{\cdot f}_{2}}^{2}\\ \frac{dTG}{dt}{\cdot f}_{2}=v_{4}{{\cdot f}_{2}}^{2}-v_{6} \end{array}\right.$$

### 2.3. Insulin Regulation of Fat Metabolism Reactions

To focus specifically on processes within adipocytes, we adopted an idealized modeling framework. We considered a scenario of minimal physical exertion, where insulin serves as the primary hormonal regulator of lipid metabolism. This allowed us to isolate the effect of insulin dynamics from confounding factors such as hormonal signals arising from physical activity. Hormones other than insulin were excluded from the model because, under the simulated conditions, insulin exerts a dominant and direct regulatory influence on both lipogenesis and lipolysis in adipose tissue.

In the model, insulin acted as an effector, which activated the transport of glucose and fatty acids into the adipocyte in reactions $v_{1}$ and $v_{2}$, respectively, and the synthesis of lipid droplet triglycerides in reaction $v_{4}$. It also inhibited the lipolysis of lipid droplet triglycerides in reaction $v_{6}$. Although the glucose release from the liver is known to decrease after meal intake due to insulin-mediated suppression of glycogenolysis and gluconeogenesis, in our model this flux was represented as a constant rate $v_{g}$. This simplification was adopted to maintain a stable baseline for plasma glucose concentration. The dependence of glucose outflow $v_{outG}$ from plasma on insulin was not included in the model, as we considered the combined effective outflows to various organs. Under resting conditions, the fraction of glucose consumed by insulin-dependent tissues (such as muscle) was relatively small compared to consumption by insulin-independent tissues (e.g., brain, kidneys). Therefore, the total glucose outflow $v_{outG}$ was assumed proportional to its plasma concentration and independent of insulin levels. Insulin activation of *i*-th reactions was modeled by a linear function $\left(1+k_{i\_act}\cdot In\right)$, reflecting the proportional dependence of the rate on the hormone concentration. Inhibition, conversely, was described by a hyperbolic function $\frac{1}{1+k_{i\_inh}\cdot In}$, accounting for the saturable suppression of lipolysis with increasing insulin level. The parameters $k_{i\_act}$ and $k_{i\_inh}$ characterize the sensitivity of the corresponding reactions to insulin.

Blood plasma insulin level is highly dependent on glucose concentration in blood plasma, while amino acids, ketone bodies, and fatty acids have a significantly smaller effect on insulin secretion [23,24]. In the suggested mathematical model, insulin secretion, dependent only on plasma glucose concentration, occurs at a rate of $v_{9}$, and its degradation occurs at a rate of $v_{10}$. The dependence of the rate of insulin secretion on blood glucose concentration is known to have an S-shaped curve [25]. To describe the rate of insulin secretion $v_{9}$, we used a phenomenological expression that allows us to reproduce the shape of the sigmoidal dependence of the rate on the concentration of glucose *Gp* in the blood plasma: $v_{9}=\frac{k_{9}}{1+e^{\frac{-\left(Gp-a\right)}{b}}}$, where $k_{9}$ characterizes the maximum rate of insulin secretion, *a* is the concentration of glucose at the inflection point, and *b* characterizes the range of glucose concentrations in which the rate of insulin secretion changes significantly. The expressions for the reaction rates $v_{i}$ in Equation (1) are presented in Table 1.

### 2.4. Model Parameterization

To parameterize the model, we used two sets of experimental data: one for healthy individuals and one for patients with type 2 diabetes. The experimental data included the dynamics of plasma metabolites after a single meal, as well as observations that the content of triglycerides in adipocytes did not change over a long period of time (1 month) in both healthy individuals [26] and patients with type 2 diabetes [16] (see Materials and Methods). The initial conditions were the concentrations of metabolites measured after an overnight fast [16,22] (Table 2), assuming that by this point the concentrations of all metabolites had reached their steady-state concentrations. In cases where the concentrations were unknown for patients with type 2 diabetes (*G3P*, *G3Pp*, *FA*, *TG*), their values were set to be the same as for healthy individuals.

**Figure 2:**
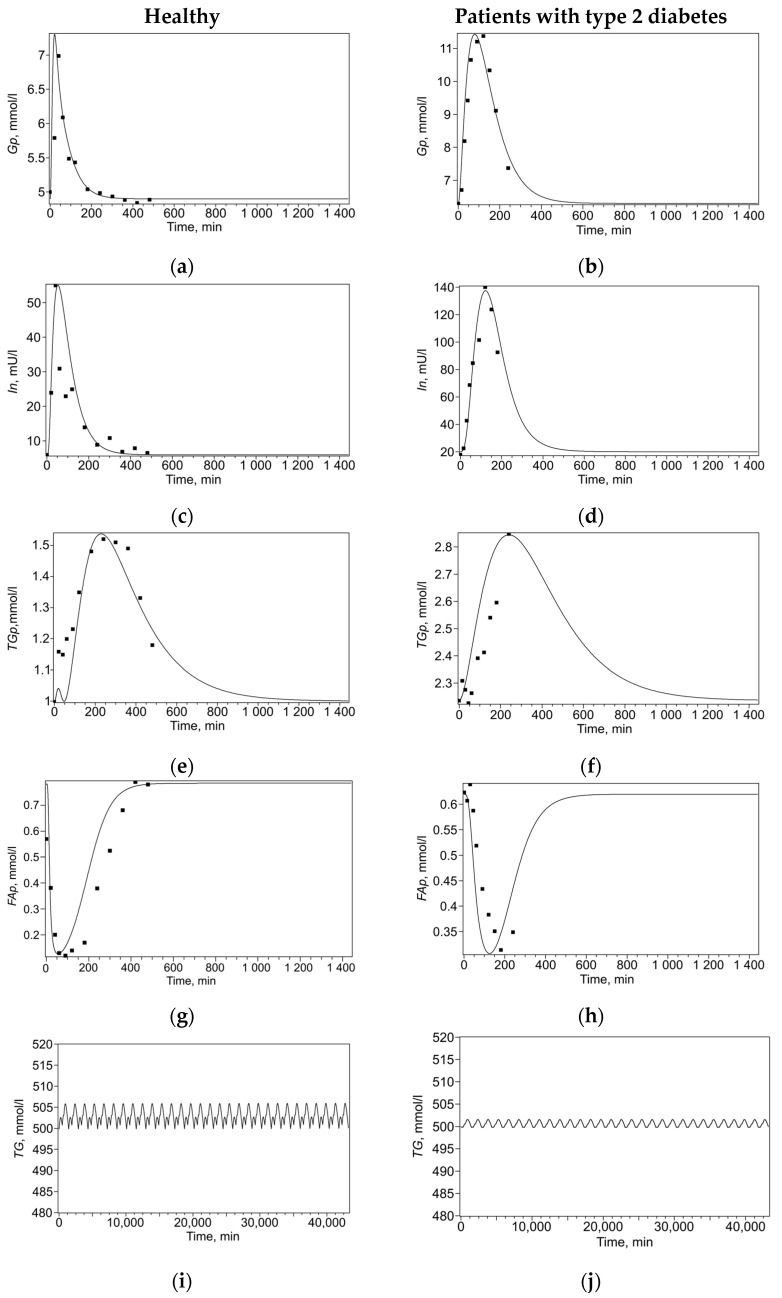
Dynamics of metabolites in healthy individuals (left column, experimental data points taken from the literature [26]) and patients with type 2 diabetes (right column, experimental data points taken from the literature [16]) after a single meal during the day (**a**–**h**) and the dynamics of triglycerides in adipocytes over the course of a month with three meals per day (**i**,**j**). Concentrations: (**a**,**b**)—glucose in plasma (*Gp*), (**c**,**d**)—insulin (*In*), (**e**,**f**)—triglycerides in plasma (*TGp*), (**g**,**h**)—fatty acids in plasma (*FAp*), (**i**,**j**)—triglycerides in adipocyte (*TG*). Points are experimental data; curves are model solutions.

The model solutions and experimental data points are presented in Figure 2. For each set of experimental data, a set of model parameters (Table 3) was found that minimized the discrepancy between the model solutions and experimental data points. To reduce ambiguity in parameter identification, fitting was performed simultaneously across the entire set of experimental curves, including both changes in plasma metabolites after a single meal and maintaining a constant triglyceride level over a month, separately for healthy subjects (Figure 2a,c,e,i) and for patients with type 2 diabetes (Figure 2b,d,f,j). This multi-curve approach constrains parameter space, as a single parameter set must reproduce multiple distinct physiological responses, thereby reducing identifiability uncertainty.

It should be noted that the constants of activation and inhibition of insulin reactions ($k_{1\_act}$, $k_{2\_act}$, $k_{4\_act}$, $k_{6\_inh}$) in patients with type 2 diabetes were significantly lower than in healthy individuals, which can be correlated with the state of insulin resistance characteristic of this disease. However, we observed that the parameter governing maximal insulin secretion ($k_{9}$) was higher for patients with type 2 diabetes than for healthy individuals, a finding consistent with experimental data showing higher insulin peaks in this population [16]. This result may appear counterintuitive, as type 2 diabetes is typically associated with a blunted insulin peak amplitude [27]. However, the patients in our dataset had concomitant obesity (mean BMI = 31) [16], and in obese individuals, including those with early-stage type 2 diabetes, compensatory hypersecretion can lead to elevated fasting and postprandial insulin levels, overriding the typical attenuation of the insulin response [28,29,30].

Thus, for each of the selected sets of parameters (for healthy individuals and patients with type 2 diabetes), the model adequately describes the dynamics of blood plasma metabolites after a single meal (Figure 2a–h), as well as the content of triglycerides in adipocytes over the course of a month with three meals a day (Figure 2i,j).

### 2.5. Triglyceride Accumulation Depending on the Number of Meals

**Figure 3:**
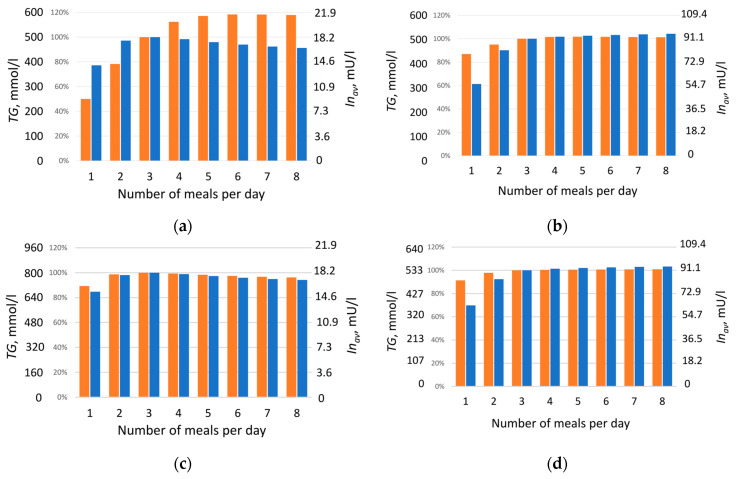
Daily average insulin concentration in plasma (blue bars), and triglyceride concentration in adipocytes (orange bars) after one month of feeding, depending on the number of daily meals under periodic insulin variation in healthy individuals (**a**) and patients with type 2 diabetes (**b**), and under constant insulin level in healthy individuals (**c**) and patients with type 2 diabetes (**d**). Daily caloric intake from lipids and carbohydrates was identical for healthy individuals and patients with type 2 diabetes, and identical across meal frequencies, amounting to 2047.7 kcal.

At the next stage, numerical experiments were conducted based on the calibrated parameters to model triglyceride accumulation in the lipid droplet of adipocyte depending on meal frequency under isocaloric conditions. In the model, food intake was represented as periodic input of lipids (*F*) and carbohydrates (*C*) into the system. Feeding regimens were considered in which the number of daily meals varied from one to eight over 12 h (720 min), with a 12 h (720 min) overnight fasting period. To compare modeling results for healthy individuals and those with type 2 diabetes, a diet was designed such that daily caloric intake was identical for all meal frequencies and for both groups, comprising 879 kcal (117 mmol) of lipids and 1168 kcal (1583 mmol) of carbohydrates. The total amount of lipids (*F*) and carbohydrates (*C*)—2047 kcal—was divided equally among the number of daily meals. Three-meal feeding was selected as the baseline, under which triglyceride content remained unchanged over the month (see Section 4). The effect of meal frequency on triglyceride (*TG*) accumulation in adipocytes was investigated, evaluating changes in their concentration at different number of daily meals, from one to eight (Figure 3, orange bars). The dynamics of all model metabolites for healthy individuals and patients with type 2 diabetes are presented in Figure S1 (Supplementary Materials), using two-meal and five-meal regimens as examples. Figure 3 and Figure S1 show that, depending on meal frequency, triglyceride (*TG*) content in adipocytes may increase, for example, under five-meal feeding (red curves in Figure S1w–z and corresponding bars in Figure 3a,b), or decrease under two-meal feeding (black curves in Figure S1w–z and corresponding bars in Figure 3a,b). This effect was weaker in patients with type 2 diabetes (Figure 3b) than in healthy individuals (Figure 3a).

Given that triglyceride accumulation, and consequently fat mass gain, is often associated with insulin level [29,31], triglyceride content was compared with the daily average insulin concentration, calculated using Formula (8) (see Section 4), for each feeding regimen (Figure 3, blue bars). As expected, patients with type 2 diabetes exhibited a several-fold higher daily average insulin level (3–5 times) than healthy individuals (Figure 3), consistent with the literature reports of hyperinsulinemia in this condition [30,32].

With increasing meal frequency, triglyceride content in adipocytes rises, and the effect is most pronounced in healthy individuals (Figure 3a, orange bars). In patients with type 2 diabetes, this dependence is less apparent (Figure 3b, orange bars), which may be explained by the presence of insulin resistance, typical for this condition. The obtained result demonstrates a clear effect: at identical daily caloric intake, constant lipid-to-carbohydrate ratio, and equal sensitivity of insulin-dependent reactions, the triglyceride content synthesized over one month (and thus fat mass) varies significantly depending on meal frequency. This indicates that fat mass accumulation is determined not only by total caloric intake but also by the frequency of nutrient delivery. Unexpectedly, a nonlinear relationship was observed between average daily insulin level and triglyceride accumulation as a function of daily meal frequency (Figure 3a). It was assumed that a reduced insulin level with more frequent meals should be accompanied by decreased triglyceride content, which generally aligns with modeling results obtained for diabetic patients (Figure 3d). However, in healthy individuals, the outcome was opposite: starting from four meals per day, insulin level decrease with increasing meal frequency, while triglyceride content rise (Figure 3a).

The observed lack of correlation between insulin level and triglyceride accumulation led to the hypothesis that differences in triglyceride accumulation relative to changes in daily average insulin level are determined specifically by the dynamics of the insulin response, rather than by the value of its daily average level. To test this hypothesis, an additional modeling experiment was conducted with a fixed insulin level corresponding to the daily average level under the respective feeding regimen. For this purpose, insulin dynamics was excluded from the model (1): d*In*/d*t* = 0, and the daily average insulin amount was set according to the values shown in Figure 3a,b. Subsequently, at constant insulin level ${In}_{av}$, concentrations of accumulated triglycerides (*TG*) were obtained for different numbers of daily meals (Figure 3c,d) in the absence of insulin dynamics. The experiment showed that in the absence of insulin fluctuations, an almost linear relationship is established between the amount of triglycerides accumulated by adipocytes over one month and insulin level (Figure 3c,d). This was true both for healthy individuals (Figure 3c) and for patients with type 2 diabetes (Figure 3d): a decrease in insulin level was accompanied by a reduction in triglyceride content, consistent with established views on insulin’s effect on lipogenesis. Therefore, our model suggests that it is the dynamics of the insulin response, not its daily average value, that accounts for the observed lack of correlation between insulin level and fat accumulation under varying meal frequencies of an isocaloric diet.

Since many reactions involved in triglyceride formation, including glucose transport into adipocytes, lipogenesis, and lipolysis, were insulin-dependent, it was important to determine the extent to which insulin affects these reactions, whether its influence was uniform or preferential toward certain reactions. To identify which lipid metabolism reactions were most sensitive to the dynamics of the insulin signal, we examined changes in metabolic fluxes upon varying the number of daily meals. The daily average reaction rates $\langle v_{i}\rangle$, calculated according to Formula (7) (see Section 4), are presented for healthy individuals in Table 4 and for patients with type 2 diabetes in Table 5. In addition to mean values, the tables indicate the ranges of variation for each rate, normalized to the maximum value of the corresponding rate. Since the considering system reached a steady state by the end of the day, starting from the first meal, the average values of $v_{001}$, $v_{002}$, $v_{8}$, $v_{7}$, $v_{10}$ are equal to the average values of $v_{01}$, $v_{02}$, $v_{6}$, $v_{2}$, $v_{9}$ respectively; therefore, only the values for the last group of reactions are given in the tables.

The data presented in Table 4 and Table 5 show that the greatest variation in daily average reaction rates upon varying meal frequency is observed for two key processes: triglyceride lipolysis in adipocytes $\langle v_{6}\rangle$ and insulin secretion $\langle v_{9}\rangle$, both in healthy individuals and in patients with diabetes. It should be noted that the rates of reactions responsible for triglyceride formation change very little with alterations in feeding regimen, in contrast to the pronounced variability in lipolysis rate, despite the general regulatory role of insulin in all these processes. Triglyceride content in adipocytes is determined by the balance between their synthesis and degradation. Since the daily amount of synthesized triglycerides was virtually independent of meal frequency (average rate $\langle v_{4}\rangle$ remained nearly constant; Table 4 and Table 5), the increase in their accumulation under more frequent feeding can be explained by a reduction in lipolysis rate $\langle v_{6}\rangle$, i.e., by a decrease in the amount of degraded triglycerides. Importantly, regardless of the chosen feeding regimen, mass balance was preserved in the calculation of metabolic fluxes: the amount of consumed substance matched the total amount excreted and accumulated (balance was determined based on molar carbon content). Thus, the observed dependence of triglyceride accumulation on meal frequency under identical daily caloric intake is not due to changes in synthesis rate, but rather to redistribution between accumulation and degradation processes, primarily through suppression of lipolysis. Analysis showed that the dynamic profile of insulin plays a key role in this effect. Its shape is determined by several parameters: amplitude and duration (width) of insulin peaks, their number, and the degree of overlap, which increases with higher meal frequency, since the next insulin response begins before the previous one has fully declined.

### 2.6. Study of the Effect of Dynamic Characteristics of the Insulin Response on Triglyceride Accumulation Using a Reduced Model

The kinetic curves of metabolites (Figure S1, Supplementary Materials) revealed that both the amplitude and width of insulin peaks varied substantially depending on meal frequency. To investigate how exactly the characteristics of the insulin profile affect triglyceride accumulation, we constructed a reduced model based on the finding of the key role of lipolysis.

**Figure 4:**
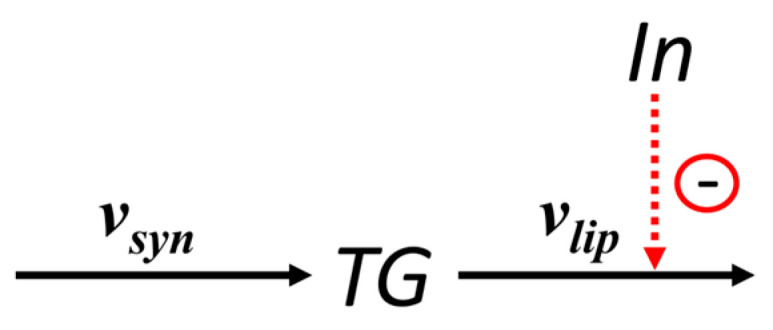
A scheme of reactions in the reduced model of triglyceride lipolysis in adipocytes. Red dashed line—inhibition of the reaction by insulin. *In*—insulin, *TG*—triglycerides in adipocytes.

Analysis of daily average metabolic reaction rates showed that upon varying meal frequency, only two key rates, triglyceride lipolysis $\langle v_{6}\rangle$ and insulin secretion, changed significantly, while the rates of all other reactions remained virtually unchanged (Table 4 and Table 5). Based on this, the original adipocyte metabolism model was reduced to a minimal lipolysis model. In the reduced version, only two processes were included: triglyceride synthesis at a constant rate $v_{syn}$ (corresponding to $v_{4}$ in the original model) and their lipolysis at rate $v_{lip}$ (corresponding to $v_{6}$ in the original model), dependent on the dynamic insulin profile (Figure 4).

This model reduction allowed focusing on the key mechanism regulating fat accumulation, suppression of lipolysis, without losing essential dynamic properties of the system. The equation describing changes in triglyceride concentration *TG* in adipocytes, according to the scheme in Figure 4, is:

(2) $$\frac{dTG}{dt}=v_{syn}-v_{lip}.$$

The general form of the dependence of the lipolysis rate on insulin concentration was set identically to that in the original model: $v_{lip}=\frac{k_{lip}}{1+k_{inh}\cdot In(t)}$, where $k_{lip}$ is the lipolysis rate in the absence of inhibition, and $k_{inh}$ is the inhibition constant for lipolysis.

In the original model, insulin dynamics depended nonlinearly on glucose concentration; therefore, when meal frequency was altered, insulin peak parameters—their amplitude, duration, and degree of overlap—changed simultaneously and interdependently. This precluded separate assessment of the contribution of each factor to lipolysis suppression and, consequently, to triglyceride accumulation. To overcome this limitation, in the reduced lipolysis model, the insulin response to food intake was defined by an analytical function (3), independent of glucose dynamics:

(3) $$In\left(t\right)=A\cdot \frac{t}{\tau }\cdot e^{-\frac{t}{\tau }}+{In}_{st}.$$

This function closely reproduces the shape of the insulin response in the original model and asymmetric profile observed in experimental data—a rapid initial rise followed by a slower decay. Importantly, this function allowed independent variation in the amplitude (A) and peak width (via parameter τ) of the insulin response, as well as setting the basal insulin level (${In}_{st}$). The peak width at A/2—$\tau_{1/2}$ was chosen to characterize peak width; for the equation of form (3), $\tau_{1/2}$ was calculated using the Lambert W-function as $\tau_{1/2}\approx 2.45\tau$. This approach enables control over individual dynamic characteristics of the insulin profile, which made it possible to study their effect on lipolysis suppression and triglyceride accumulation in adipocytes.

Instead of periodic food intake (as in the original model), the reduced model considered the corresponding sequence of insulin concentration peaks, defined by a formula of the form (3):

(4) $$In(t)=\sum_{i}A_{i}(t)\frac{\left(t-T_{i}\right)}{\tau }e^{-\frac{\left(t-T_{i}\right)}{\tau }}+{In}_{st},$$

where $T_{i}$—are the onset times of the insulin response, $A_{i}\left(t\right)=0$ for $t<T_{i}$, $A_{i}\left(t\right)=A$ for $t\geq T_{i}$, A is the peak amplitude corresponding to the given number of insulin responses. Taking Formula (4) into account, the daily average insulin level can be calculated as

(5) $$In_{av}=\frac{1}{T}\sum_{i}\int_{T_{i}}^{T}A\frac{\left(t-T_{i}\right)}{\tau }e^{-\frac{\left(t-T_{i}\right)}{\tau }}dt+{In}_{st},$$

where T = 1440 min (1 day).

If there is no peak overlap, the average insulin level can be estimated as

(6) $${In}_{av}\approx \frac{1}{T}\cdot n\cdot A\cdot \tau \cdot e+{In}_{st},$$

where n—number of peaks.

**Figure 5:**
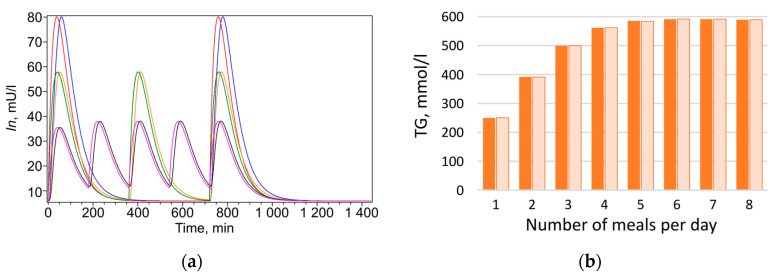
Comparison of the calculation results of the original and reduced models. (**a**) Insulin dynamics over 1 day with two, three, and five meals in the original model (blue, orange, and black curves, respectively) and in the reduced model (red, green, and purple curves, respectively); (**b**) Adipocyte triglyceride content after 1 month in the original (orange bars) and reduced (light orange bars) models with different numbers of meals; $v_{syn}=0.017$; $k_{lip}=0.07$; $k_{inh}=0.29$. The insulin response characteristics are presented in Table 6.

The parameters of the reduced model ($v_{syn}$, $k_{lip}$, $k_{inh}$) and insulin response parameters (A, $\tau_{1/2}$, and ${In}_{st}$) were not directly inherited from the full model, as the reduction process eliminates all non-essential reactions. Instead, they were calibrated such that the solutions of the reduced model, specifically the dynamics of insulin concentration (Figure 5a) and the final triglyceride content after one month (Figure 5b), closely matched those of the original model across different feeding regimens.

**Table 6:** Characteristics of the insulin response in a reduced model.

n (number of peaks)	1	2	3	4	5	6	7	8
A (peak amplitude, mU L^–1^)	280.5	202	141	103.7	81	64.7	53.5	45.4
$\tau_{1/2}$ (peak half-width, min)	79.9	95.6	102.9	104.1	105.4	107.3	109.0	110.5
$\frac{T}{2(n-1)}\;$ (interval between peaks, min)	–	720	360	240	180	144	120	103

Thus, solutions of the reduced model (2–6) under variations in parameters A and $\tau_{1/2}$ adequately reproduce the behavior of the full model in response to changes in the insulin profile, enabling its use for analyzing the influence of individual dynamic parameters on triglyceride content in adipocytes.

To assess the individual contribution of amplitude (A), width ($\tau_{1/2}$) of insulin peaks, their number (n), and the intervals between them (partial peak overlap may occur with short intervals) to triglyceride accumulation in adipocytes, a series of numerical experiments was conducted using the reduced lipolysis model, analyzing triglyceride accumulation over one month.

To avoid peak overlap when varying amplitude and width under periodic insulin delivery, in subsequent numerical experiments the peak width was reduced ($\tau_{1/2}=49$ min). The lipolysis rate constant ($k_{lip}$) was adjusted so that, at the selected peak width, triglyceride content in adipocytes remained unchanged by the end of the month under thrice-daily insulin delivery, consistent with the behavior of the original model. For convenience of comparison, triglyceride values obtained in different numerical experiments were normalized to a chosen reference value defined as the triglyceride content at the end of the month under thrice-daily insulin delivery.

**Figure 6:**
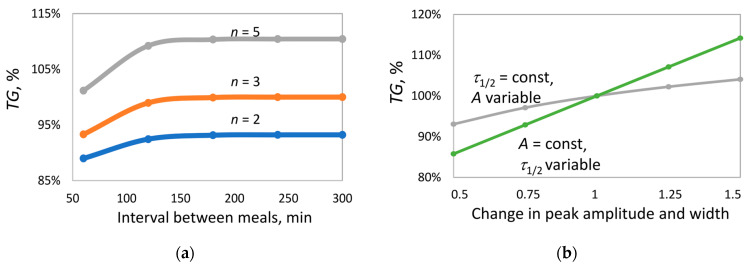
The effect of dynamic characteristics of the insulin response on triglyceride (*TG*) content in adipocytes at the end of the month. (**a**) Effect of interval length between peaks at different numbers n of insulin delivery and $\tau_{1/2}=49$. For n=2 (blue curve), A=211.5; for n=3 (orange curve), A=141; for n=5 (gray curve), A=84. (**b**) Effect of amplitude (*A*) (gray curve) and width ($\tau_{1/2}$) (green curve) of the insulin response under thrice-daily insulin delivery, with reference values *A* = 141, $\tau_{1/2}=49$ at n=3. The abscissa shows the factor by which *A* and *τ*_1/2_ were varied relative to reference values. $v_{syn}=0.017$; $k_{lip}=0.056$; $k_{inh}=0.29$.

In the first series of experiments, the effect of the interval between insulin peaks (Figure 6a) was investigated under two-, three-, and five-fold insulin delivery. The interval was varied from 60 to 300 min. At long intervals (180–300 min), when peak overlap is nearly absent, triglyceride accumulation by the end of the month increases with increasing number of peaks n. In contrast, at short intervals (60 min), strong peak overlap occurs, leading to a reduction in triglyceride content by 2%, 7%, and 10% for n = 2, 3, and 5, respectively. Nevertheless, even under maximum overlap, triglyceride accumulation at *n* = 5 remains higher than at n = 2. Importantly, in all cases, with the selected parameters, the average insulin level was identical, excluding the influence of this parameter on the outcome.

In the next series of experiments, under thrice-daily insulin delivery (interval 300 min, no peak overlap; Figure 6b), the effects of amplitude and peak width on triglyceride accumulation were compared. According to Formula (6), altering either parameter results in a proportional change in the average insulin level (${In}_{av}$). To isolate the effect of each parameter, they were varied independently, and both were scaled by the same factor, so that ${In}_{av}$ increased to the same extent in both cases.

At fixed peak width, increasing amplitude led to ~11% increase in *TG* content. In contrast, at fixed amplitude, increasing peak width resulted in ~30% increase in *TG* content. This suggests that peak width exerts a significantly stronger influence on lipid balance than amplitude, despite identical changes in average insulin level.

## 3. Discussion

The aim of this study was to test the hypothesis that meal frequency, at constant daily caloric intake, may influence triglyceride accumulation in adipocytes. For this purpose, a mathematical model of carbohydrate–lipid metabolism was developed, capturing the dynamics of key metabolites in blood plasma and adipocytes, while allowing variation in meal number, frequency, and macronutrient composition. The model accounts for the two-stage process of digestion: nutrient entry into the gastrointestinal tract and subsequent transport of glucose and fatty acids into blood plasma. This enabled an adequate description of glucose, insulin, and fatty acid dynamics following a single meal. Although the accuracy of describing multiple meals (compared to single meals) is somewhat reduced due to simplifications in nutrient absorption kinetics, the model is sufficient for analyzing qualitative patterns and identifying potential mechanisms by which meal regimen affects lipid metabolism. Since the primary focus was on processes within the adipocyte, the model includes key insulin-regulated reactions of triglyceride synthesis and degradation, with insulin serving as the primary hormonal regulator of lipid metabolism in adipose tissue [12]. Other regulatory factors (leptin, glucagon, etc.) are not included in the model, representing a simplification that allows isolation of insulin’s contribution. The model is limited to blood plasma and adipocytes, justified by its focus on intracellular fat storage processes.

During model parameterization for patients with type 2 diabetes, reaction rate constants characterizing insulin’s effects were found to be smaller compared to healthy individuals, reflecting the clinical picture of insulin resistance and enabling adequate reproduction of metabolic profiles in these patients.

The key modeling result is that, at constant daily caloric intake and macronutrient ratio, meal frequency substantially affects triglyceride content in adipocytes. With infrequent feeding (1–2 meals per day), fat mass decreases, whereas with frequent feeding (5–8 meals per day), it increases. This effect is most pronounced in healthy individuals and weaker in patients with type 2 diabetes.

At first glance, fat accumulation should correlate with average insulin level, since insulin stimulates triglyceride synthesis and suppresses lipolysis. However, analysis revealed a nonlinear relationship between daily average insulin concentration and triglyceride accumulation in healthy individuals: as meal frequency increases, insulin level initially rises and then declines, while triglyceride content monotonically increases. In contrast, patients with type 2 diabetes exhibit a more direct relationship, consistent with their chronic hyperinsulinemia and high basal stimulation of lipogenesis.

This discrepancy led to the hypothesis that it is not the average insulin level but its dynamic profile, amplitude, duration, and degree of peak overlap, that plays a decisive role in regulating lipid metabolism. This was confirmed by simulations under constant (steady-state) insulin level: in this case, the relationship between insulin and triglyceride accumulation became linear, consistent with classical views of its action.

Further analysis showed that the rate of triglyceride synthesis (reaction $v_{4}$) remains virtually unchanged with varying meal frequency, whereas the rate of lipolysis ($v_{6}$) significantly decreases with more frequent feeding. Thus, our model indicates fat accumulation is driven not by enhanced lipogenesis but by suppressed lipolysis, and this reaction is most sensitive to changes in the insulin profile. This observation is consistent with the literature data. Insulin suppresses lipolysis by inhibiting key enzymes, such as adipose triglyceride lipase and hormone-sensitive lipase [33]. Although insulin also stimulates glucose transport into adipocytes via GLUT4 [34], experiments with GLUT4-deficient mice show that this does not lead to significant changes in fat accumulation [35], indicating higher regulatory sensitivity of lipolysis compared to glucose transport.

To investigate the contribution of individual dynamic characteristics of the insulin response, a reduced model was developed, in which the insulin profile was defined by an analytical function. On one hand, the reduced model qualitatively yielded the same results as the full model. On the other hand, it allowed independent variation in intervals between meals, amplitude, and width of the insulin response peak.

Analysis of results obtained with the reduced model showed that both increasing the amplitude and increasing the width of the insulin peak enhance lipolysis suppression and promote triglyceride accumulation. However, increasing peak width exerts a stronger effect than an equivalent increase in amplitude. In other words, the duration of insulin action (peak width) carries greater weight in suppressing lipolysis than its peak value. The observed model effects find physiological parallels in known metabolic responses. It is known that lowering the blood insulin level correlates with a reduction in fat stores [36]. Decreasing the peak amplitude corresponds to reduced food intake, for example, under moderate caloric restriction. Decreasing the peak width may be induced by physical activity after meals, for instance, walking, which accelerates metabolism and shortens the duration of the insulin response. According to model results, it can be expected that physical activity would produce a greater effect than caloric restriction at the same average insulin level.

Such a difference in the impact of amplitude versus peak width can be explained by the nonlinear nature of inhibition: since lipolysis suppression in the considered models follows a hyperbolic function, even a modest increase in insulin exposure duration can lead to a substantial reduction in triglyceride breakdown rate, particularly in the saturation region of the function, where the reaction becomes less sensitive to increasing concentration but highly dependent on exposure duration. The hyperbolic dependence of lipolysis on insulin concentration is consistent with known experimental data [37,38]. Modeling results show that it is precisely this form of inhibitory function that gives rise to complex dynamic effects that cannot be predicted based on the average hormone level.

Thus, the obtained results indicate that the meal regimen, even under isocaloric conditions, may significantly affect the lipid metabolism through the formation of a specific insulin profile. Frequent feeding, despite lower insulin peaks compared to less frequent meals, leads to more sustained suppression of lipolysis and consequently greater triglyceride accumulation, particularly when significant peak overlap is absent. This effect is most pronounced in healthy individuals and attenuated under insulin resistance.

While some experimental studies have reported that reduced meal frequency is associated with lower body weight gain or improved body composition [39], the overall evidence remains inconsistent. Meta-analyses and reviews highlight substantial heterogeneity in study designs, participant characteristics, and dietary interventions, making it difficult to draw firm conclusions about the effect of meal frequency on fat accumulation [8,40]. This inconsistency likely arises from the multitude of confounding factors, including total caloric intake, macronutrient composition, physical activity, and individual metabolic variability, that influence energy balance in real-world settings. Our model, by contrast, isolates the specific role of insulin dynamics under strictly controlled isocaloric conditions. By focusing on the adipocyte and eliminating other physiological variables, we were able to demonstrate a clear mechanistic link: fewer meals lead to less triglyceride accumulation primarily because they result in shorter, less overlapping insulin peaks, thereby allowing greater lipolysis. This finding provides a potential explanation for why, in some clinical and observational studies, infrequent eating is associated with reduced fat gain—not necessarily due to total calories, but due to the temporal pattern of nutrient delivery and its downstream hormonal consequences.

It should be noted that the implications of these findings for in vivo physiology should be interpreted with great caution. The model intentionally excludes explicit representations of the dynamics of the liver and skeletal muscle metabolism; their contributions are reduced to effective fluxes that reflect overall systemic effects rather than organ-specific dynamics. This simplification was necessary to isolate the primary mechanism under study: how insulin dynamics regulate lipid accumulation in adipocytes. This is a fundamental limitation of the model. In complex biological systems, separating the contributions of individual components is inherently challenging. The dynamic, interconnected reactions occurring in the liver and muscle are not reflected in our current model. Nevertheless, the model successfully reproduces the experimental dynamics of key carbohydrate-lipid metabolites in blood plasma for both healthy individuals and patients with type 2 diabetes after a single meal (Figure 2).

Crucially, the conclusions drawn from the model at this stage of the study apply strictly to processes occurring within the adipocyte. We identified a non-intuitive, dominant role for adipocyte lipolysis: its nonlinear suppression by insulin renders fat accumulation more sensitive to the temporal profile of insulin than to its average daily concentration. To evaluate the impact at the whole-body level, the contribution of dynamic metabolic fluxes from the liver and skeletal muscle must be incorporated. The extent of their influence on the adipocyte-specific mechanism identified here may be substantial or relatively minor; however, this remains unknown without an integrated multicomponent model. Nevertheless, by first isolating and quantifying the dominant mechanism in adipocytes (suppression of lipolysis), we have laid an important foundation for a further, more targeted approach to expand the model to include the liver and muscle compartments and identify their contribution to the dynamics of triglyceride accumulation.

The conclusions of this work emphasize the importance of a dynamic approach to analyzing hormonal regulation of metabolism and may be relevant for developing personalized dietary recommendations that consider not only the composition and caloric content of the diet but also the temporal pattern of meals.

## 4. Materials and Methods

### 4.1. Experimental Data for Model Parameterization

The model was parameterized using plasma metabolite dynamics data: glucose, insulin, triglycerides, and free fatty acids from healthy individuals [26] and patients with type 2 diabetes mellitus [16]. The body mass index (BMI) of healthy subjects was 22.5 (normal weight), whereas for type 2 diabetic patients it was 31 (class I obesity). To simulate a single meal intake, the fat-to-carbohydrate ratio (*F*:*C*) for healthy individuals was taken as 0.4:0.52 [26], and for type 2 diabetic patients as 0.3:0.55 [16]. When simulating three meals per day, the daily caloric intake was approximately 2500 kcal for healthy subjects [26] and approximately 2300 kcal for diabetic patients [16]. According to experimental data [16,26], the body weight for both healthy subjects and diabetic patients remained stable during the one-month observation period. The absence of body weight change was interpreted by us as the absence of changes in adipose tissue mass, which, within our model, corresponds to no change in triglyceride concentration within adipocytes. At the same time, we assumed that the number of adipocytes in adults remains essentially constant [21], and changes in adipose tissue mass are primarily due to fluctuations in lipid droplet volume (triglyceride content) within existing cells.

### 4.2. Caloric-to-Molar Conversion of Nutrients

To correlate the amounts of dietary fats and carbohydrates, given in kilocalories, with the level of corresponding metabolites measured in mmol L^−1^, the mathematical model included a conversion of nutrient calories into equivalent millimole quantities. This conversion was based on the most common representatives of fats and carbohydrates, tripalmitin and glucose, respectively. Glucose is a constituent of the most frequently consumed polysaccharides, starch and glycogen. Tripalmitin, as a typical triglyceride, is metabolized into glycerol and three molecules of palmitic acid, one of the most abundant fatty acids in the human body and in the diet [41]. The results of the conversion are presented in Table 7. The calculated dietary intakes were subsequently used for the parameterization of the model.

### 4.3. Calculation of the Average Rate and the Average Metabolite Level

To compare the magnitudes of metabolic fluxes during periodic food intake, the average rate of change in metabolite concentration over a day was used:

(7) $$\langle v_{i}\rangle =\frac{1}{T}\int_{0}^{T}v_{i}(t)dt,$$

where $v_{i}(t)$ is the rate of change in the concentration of the *i*-th metabolite during the period T=1440 min (1 day).

The average insulin level was estimated from the net daily flux, accounting for both secretion and degradation over one day:

(8) $${In}_{av}=\frac{1}{T}\int_{0}^{T}In\left(t\right)dt,$$

where In(t) is the change in insulin concentration during the period T=1440 min (1 day).

### 4.4. Numerical Simulations and Modeling Periodic Meal Intake

For numerical calculations, the software DBSolveOptimum2020 [42] was used with the model implementation provided in Supplementary File S1. This software solves the systems of ordinary differential equations and allows for a parameter identification of the model based on experimental data.

The intake of food into the digestive system was modeled as follows. At the initial time point, the concentrations of carbohydrates *C* and fats *F* in the digestive system were set to zero. Meal intake was defined as a discrete increment of variables *C* and *F*, corresponding to the amount of carbohydrates and fats consumed per meal. This quantity was calculated based on the specified feeding regimen (e.g., one-third of the daily norm for a three-meal regimen).

Upon entering the digestive system, macronutrients *C* and *F* were converted into *Cs* and *Fs*, representing the accumulated mass of undigested carbohydrates and fats within the digestive system, which were then gradually consumed in reactions v_01_ and v_02_. Thus, variables *Cs* and *Fs* are key for modeling the digestion process.

Subsequent meals were modeled analogously: at predefined time intervals (e.g., every 4 h), new portions of macronutrients were added to the current values of *C* and *F*. By the end of the day (after the last meal and prior to the overnight fast), the values of *C*, *F*, *Cs*, and *Fs* were reset to zero, reflecting the complete emptying of the gastrointestinal tract.

An example of the dynamics of variables *C* and *F* for 2-meal and 5-meal regimens in healthy individuals and patients with type 2 diabetes is presented in Figure S1a–d (Supplementary Materials).

## 5. Conclusions

The constructed model allows us to infer which characteristics of meal regimen may influence changes in triglyceride content within adipocytes under isocaloric conditions and constant macronutrient ratios in both healthy individuals and patients with type 2 diabetes. Our simulations indicate that the number of daily meals affects triglyceride accumulation primarily through its impact on the dynamic profile of insulin secretion. Crucially, this effect is mediated not by changes in average insulin concentration, but by alterations in the amplitude, duration (width), and degree of overlap of insulin peaks. Among all dynamic parameters, peak width, reflecting the duration of insulin exposure, was found to exert the strongest influence on triglyceride accumulation, significantly outweighing the effect of peak amplitude. Thus, our model supports the hypothesis that dietary guidelines should account for meal patterns, as the temporal structure of nutrient delivery can profoundly influence metabolic outcomes even when total energy intake remains unchanged.

## Figures and Tables

**Table 1:** Expressions for reaction rates in the model Equation (1).

Reaction Rate	Reaction Rate Expression	Description
$v_{001}$	$k_{001}\cdot C$	The entry of carbohydrates from food into the digestive system
$v_{002}$	$k_{002}\cdot F$	The entry of dietary fats into the digestive system
$v_{01}$	$k_{01}\cdot Cs$	The release of glucose into the bloodstream after the digestion of carbohydrates
$v_{02}$	$k_{02}\cdot Fs$	The release of triglycerides into the blood after the digestion of fats
$v_{1}$	$k_{1}\cdot Gp\cdot \left(1+k_{1\_act}\cdot In\right)$	Glucose transport into adipocytes and glycolysis to glyceraldehyde-3-phosphate, activated by insulin
$v_{2}$	$k_{2}\cdot TGp\cdot \left(1+k_{2\_act}\cdot In\right)$	Lipolysis of triglycerides on the vascular wall and transport of fatty acids into the adipocyte, activated by insulin
$v_{3}$	$k_{3}\cdot G3P$	Outflow of glyceraldehyde-3-phosphate for other metabolic needs
$v_{4}$	$k_{4}\cdot G3P\cdot FA\cdot (1+k_{4\_act}\cdot In)$	Insulin-activated resynthesis of triglycerides from fatty acids and glyceraldehyde-3-phosphate in adipocytes
$v_{6}$	$\frac{k_{6}}{1+k_{6\_inh}\cdot In}$	Insulin-inhibited lipolysis of triglyceride lipid droplets in adipocytes
$v_{7}$	$k_{7}\cdot Glr$	Glycerol outflow after lipolysis of triglycerides in the blood
$v_{8}$	$k_{8}\cdot FAp$	Outflow of fatty acids from blood plasma to other organs
$v_{9}$	$\frac{k_{9}}{1+e^{\frac{-\left(Gp-a\right)}{b}}}$	Insulin secretion depending on blood glucose concentration
$v_{10}$	$k_{10}\cdot In$	Insulin degradation
$v_{tg}$	*const*	Release of triglycerides into the blood from the liver
$v_{g}$	*const*	Glucose release into the blood from the liver
$v_{outG}$	$k_{outG}\cdot Gp$	Outflow of glucose from blood plasma to other organs

**Table 2:** Metabolite concentrations after overnight fasting.

Designation in the Model	Healthy [22]	Patients with Type 2 Diabetes [16]	Units
*Cs*	0.00	0.00	mmol
*Fs*	0.00	0.00	mmol
*Gp*	4.90	6.30	mmol·L^–1^
*TGp*	1.00	2.24	mmol·L^–1^
*G3P*	0.17	0.17 *	mmol·L^–1^
*FA*	0.57	0.57 *	mmol·L^–1^
*TG*	500.00	500.00 *	mmol·L^–1^
*FAp*	0.78	0.62	mmol·L^–1^
*G3Pp*	0.05	0.05 *	mmol·L^–1^
*In*	6.00	20.00	mU L^–1^

* Value was set to be the same as for healthy individuals.

**Table 3:** Values of model parameters for healthy individuals and patients with type 2 diabetes.

Constant	Healthy	Patients with Type 2 Diabetes	Units
$k_{001}$	0.0181	0.0156	min^–1^
$k_{002}$	0.0066	0.0068	min^–1^
$k_{01}$	0.0395	0.0156	min^–1^
$k_{02}$	0.0078	0.0068	min^–1^
$v_{g}$	5.2429	2.4248	mmol min^–1^
$k_{1\_act}$	0.0100	0.0001	L mU^–1^
$k_{1}$	0.1924	0.0187	min^–1^
$k_{outG}$	0.0100	0.0582	min^–1^
$v_{tg}$	0.2436	0.1489	mmol min^–1^
$k_{2}$	0.0460	0.0133	min^–1^
$k_{2\_act}$	0.0100	0.0001	L mU^–1^
$k_{7}$	0.9744	0.5958	min^–1^
$k_{4\_act}$	0.0100	0.0001	L μU^–1^
$k_{4}$	0.0097	0.0016	L μU^–1^
$k_{3}$	3.8620	0.2017	min^–1^
$k_{6}$	1.3600	0.3778	mmol min^–1^
$k_{6\_inh}$	0.2914	0.0105	L μU^–1^
$k_{8}$	0.3784	0.3021	min^–1^
$k_{9}$	2.9001	6.0292	mU·L^−1^·min^–1^
$k_{10}$	0.0214	0.0243	min^–1^
$f_{1}$	5.00	5.00	L
$f_{2}$	15.60	32.00	L
a	6.5000	10.3489	mmol^–1^
b	0.5209	1.6621	L mmol^–1^

**Table 4:** Daily average reaction rates $\langle v_{i}\rangle$ (mmol·L^–1^·day^–1^) depending on meal frequency in the simulation of feeding for healthy individuals.

Daily Average Reaction Rates	Number Of Meals Per Day								Range of Variations
	1	2	3	4	5	6	7	8	
$\langle v_{01}\rangle$	1583.2	1583.2	1583.2	1583.2	1583.2	1583.2	1583.2	1583.2	0.00%
$\langle v_{02}\rangle$	117.3	115.7	116.1	116.2	116.3	116.4	116.4	116.4	1.36%
$\langle v_{1}\rangle$	8752.1	8754.4	8752.9	8750.9	8749.3	8748.2	8747.5	8747.0	0.08%
$\langle v_{2}\rangle$	468.0	466.4	466.9	467.0	467.1	467.1	467.2	467.2	0.34%
$\langle v_{3}\rangle$	17,036.2	17,042.3	17,038.9	17,034.8	17,031.5	17,029.3	17,027.8	17,026.8	0.09%
$\langle v_{4}\rangle$	468.0	466.4	466.9	467.0	467.1	467.1	467.2	467.2	0.34%
$\langle v_{6}\rangle$	598.0	522.8	466.9	434.8	422.6	419.4	419.5	420.7	29.87%
$\langle v_{9}\rangle$	290.7	365.8	376.5	370.3	361.5	353.7	347.9	343.7	22.77%

**Table 5:** Daily average reaction rates $\langle v_{i}\rangle$ (mmol·L^–1^·day^–1^) depending on meal frequency in the simulation of feeding for patients with type 2 diabetes.

Daily Average Reaction Rates	Number of Meals Per Day								Range of Variations
	1	2	3	4	5	6	7	8	
$\langle v_{01}\rangle$	1583.2	1583.1	1583.1	1583.1	1583.1	1583.1	1583.1	1583.2	0.01%
$\langle v_{02}\rangle$	117.2	115.1	115.6	115.8	115.9	116.0	116.0	116.1	1.84%
$\langle v_{1}\rangle$	1245.8	1246.3	1246.0	1245.7	1245.7	1245.8	1245.9	1246.0	0.05%
$\langle v_{2}\rangle$	331.7	329.6	330.1	330.3	330.4	330.5	330.5	330.6	0.65%
$\langle v_{3}\rangle$	2159.9	2163.1	2161.8	2161.1	2161.0	2161.2	2161.3	2161.5	0.15%
$\langle v_{4}\rangle$	331.7	329.6	330.1	330.3	330.4	330.5	330.5	330.6	0.65%
$\langle v_{6}\rangle$	401.3	355.9	330.1	322.0	321.0	321.7	322.6	323.4	20.00%
$\langle v_{9}\rangle$	1524.7	2241.7	2485.3	2529.5	2549.4	2566.5	2580.1	2590.4	41.14%

**Table 7:** Conversion of the amount of fats and carbohydrates in calories consumed per day into the number of moles for healthy individuals and patients with type II diabetes.

Nutrient	Molecular Mass, g mol^–1^	Energy Density, kcal g^–1^	Group	Amount Consumed per Day, kcal	Amount Consumed per Day, mmol
Glucose	180	4.1	healthy	1168 [26]	1583.6
			patients with type 2 diabetes	1246 [16]	1689
Tripalmitin	806	9.3	healthy	920 [26]	122.7
			patients with type 2 diabetes	680 [16]	91

## Data Availability

Mathematical models used for numerical calculations are available as Supplementary File S1.

## Supplementary Materials

The following supporting information can be downloaded at https://www.mdpi.com/article/10.3390/ijms262411805/s1.
