# Supplementary material for: Triglyceride Accumulation in Adipocytes Modulated by Insulin Dynamics
Source: Int J Mol Sci. 2025 Dec 6;26(24):11805. doi: 10.3390/ijms262411805 (PMC12732596; doi:10.3390/ijms262411805)
Supplement: Supplementary file 1 [file ijms-26-11805-s001.zip › Figure S1.pdf]

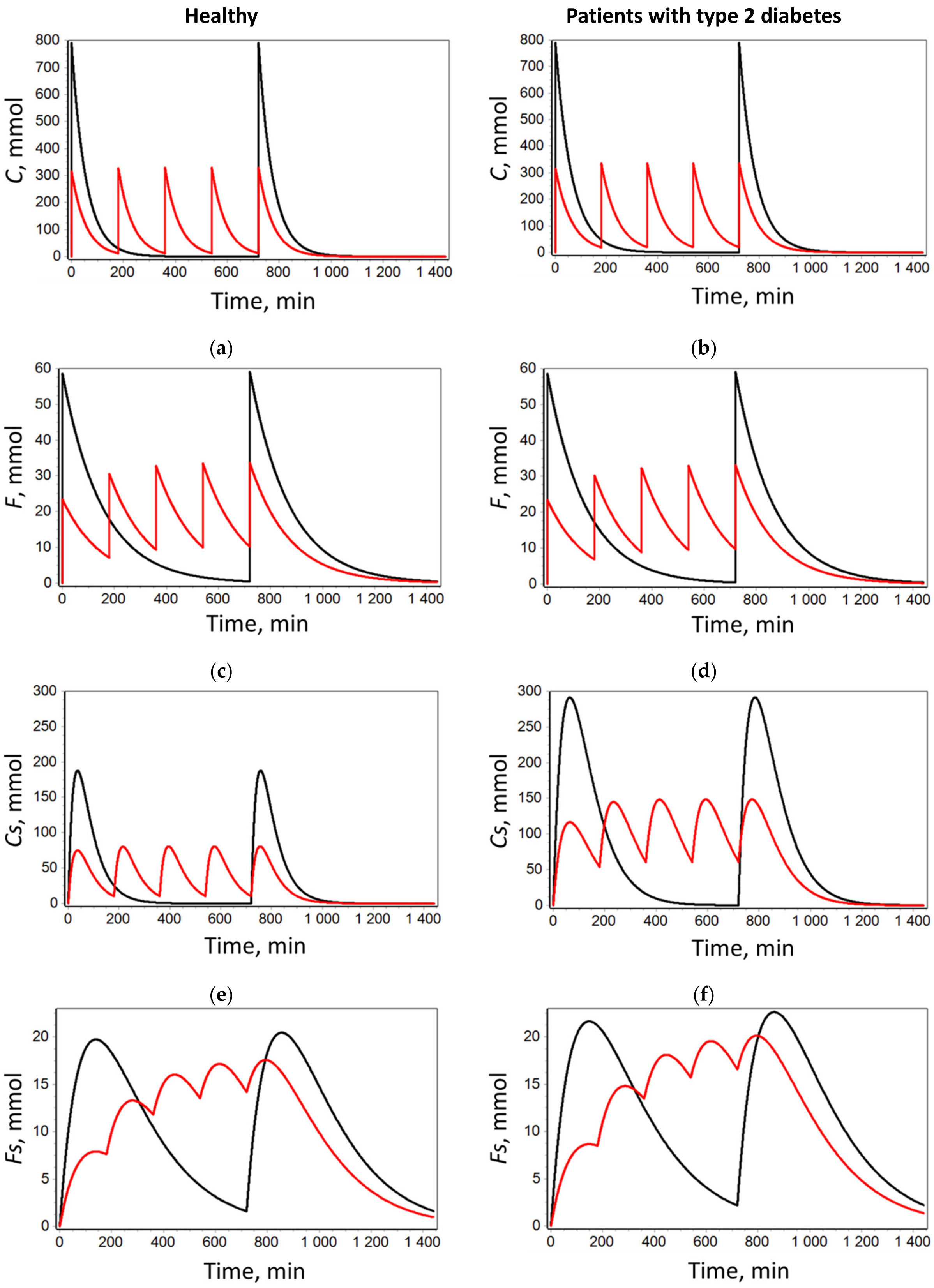
Healthy
Patients with type 2 diabetes
C, mmol
F, mmol
Cs, mmol
Fs, mmol
Time, min

(a) (b)
(c) (d)
(e) (f)
(g) (h)

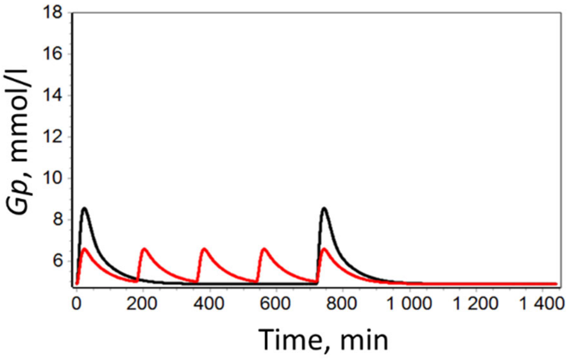
Gp, mmol/l
18
16
14
12
10
8
6
0
200
400
600
800
1 000
1 200
1 400
Time, min

(**i**)

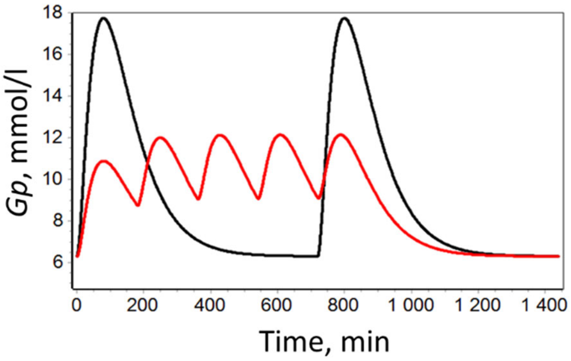
Gp, mmol/l
18
16
14
12
10
8
6
0
200
400
600
800
1 000
1 200
1 400
Time, min

(**j**)

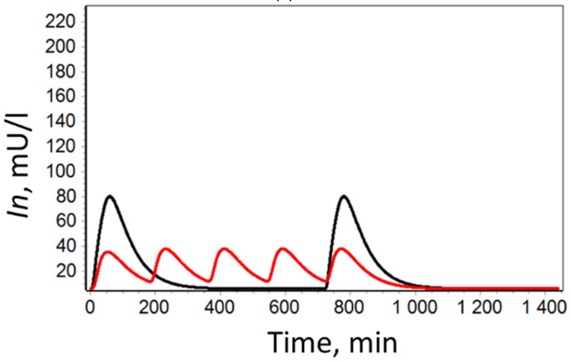
In, mU/l
220
200
180
160
140
120
100
80
60
40
20
0
200
400
600
800
1 000
1 200
1 400
Time, min

(**k**)

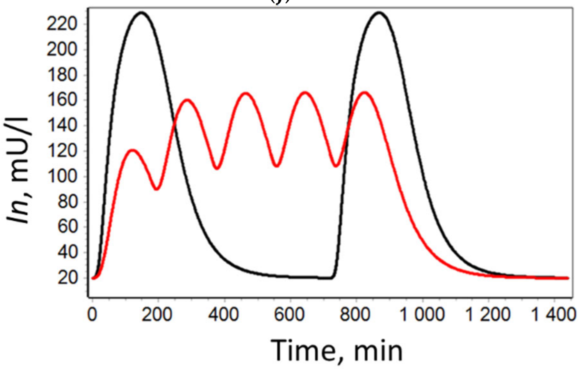
In, mU/l
220
200
180
160
140
120
100
80
60
40
20
0
200
400
600
800
1 000
1 200
1 400
Time, min

(**l**)

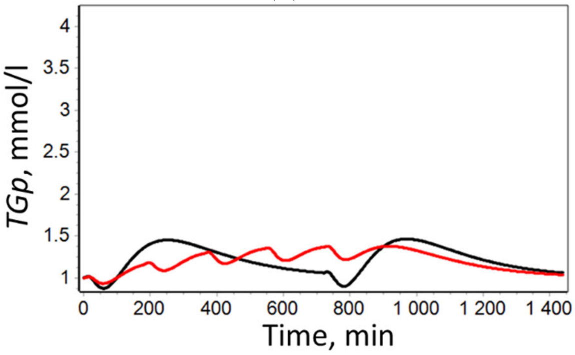
TGp, mmol/l
4
3.5
3
2.5
2
1.5
1
0
200
400
600
800
1 000
1 200
1 400
Time, min

(**m**)

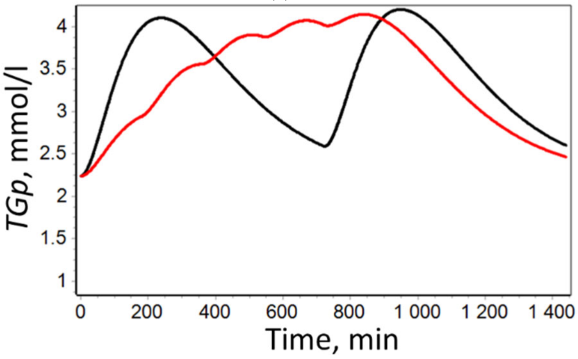
TGp, mmol/l
4
3.5
3
2.5
2
1.5
1
0
200
400
600
800
1 000
1 200
1 400
Time, min

(**n**)

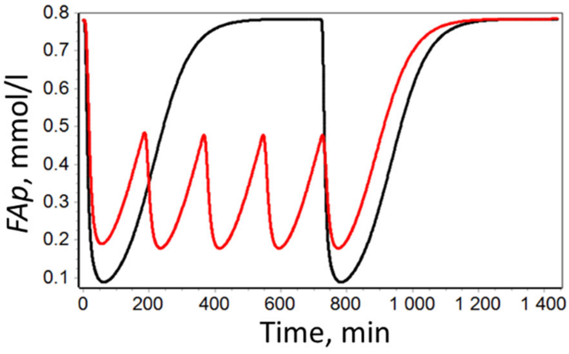
FAp, mmol/l
0.8
0.7
0.6
0.5
0.4
0.3
0.2
0.1
0
200
400
600
800
1 000
1 200
1 400
Time, min

(**o**)

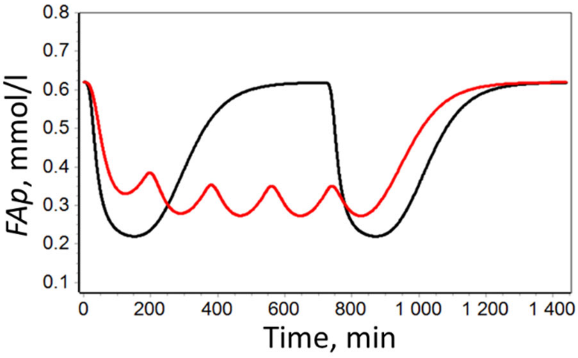
FAp, mmol/l
0.8
0.7
0.6
0.5
0.4
0.3
0.2
0.1
0
200
400
600
800
1 000
1 200
1 400
Time, min

(**p**)

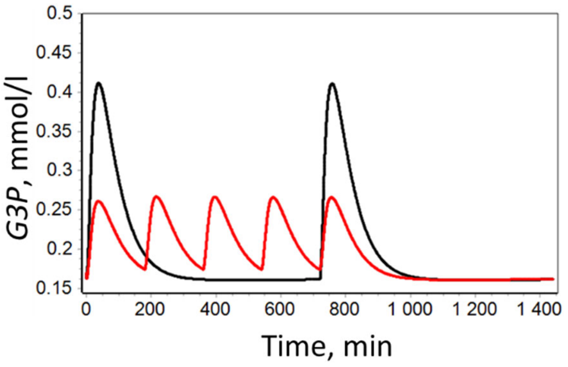
G3P, mmol/l
0.5
0.45
0.4
0.35
0.3
0.25
0.2
0.15
0 200 400 600 800 1 000 1 200 1 400
Time, min

(**q**)

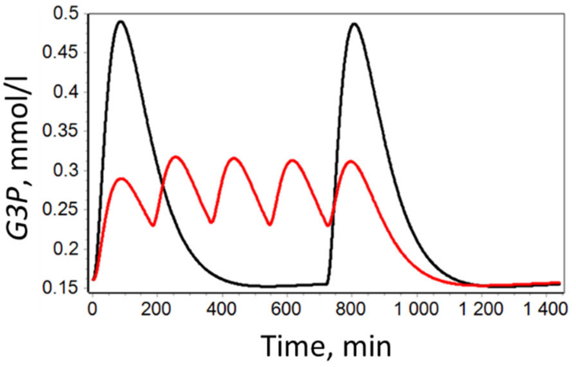
G3P, mmol/l
0.5
0.45
0.4
0.35
0.3
0.25
0.2
0.15
0 200 400 600 800 1 000 1 200 1 400
Time, min

(**r**)

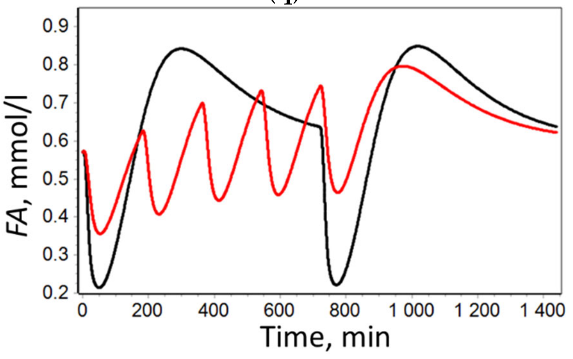
FA, mmol/l
0.9
0.8
0.7
0.6
0.5
0.4
0.3
0.2
0 200 400 600 800 1 000 1 200 1 400
Time, min

(**s**)

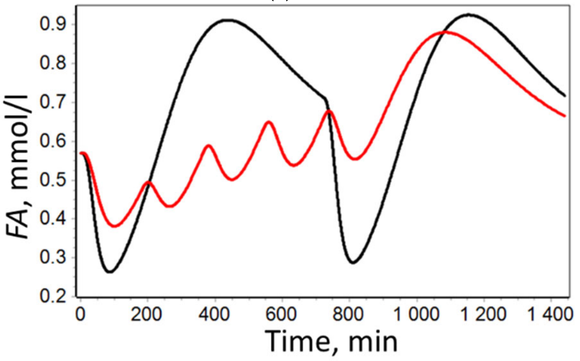
FA, mmol/l
0.9
0.8
0.7
0.6
0.5
0.4
0.3
0.2
0 200 400 600 800 1 000 1 200 1 400
Time, min

(**t**)

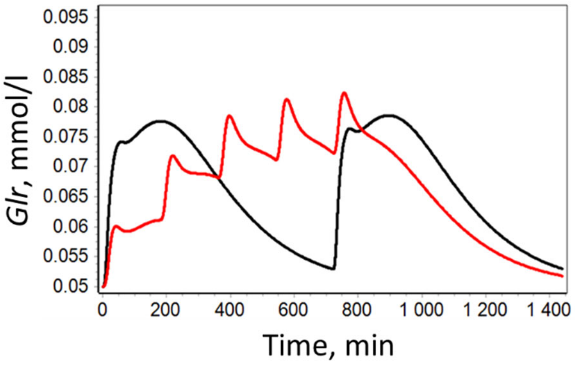
Glr, mmol/l
0.095
0.09
0.085
0.08
0.075
0.07
0.065
0.06
0.055
0.05
0 200 400 600 800 1 000 1 200 1 400
Time, min

(**u**)

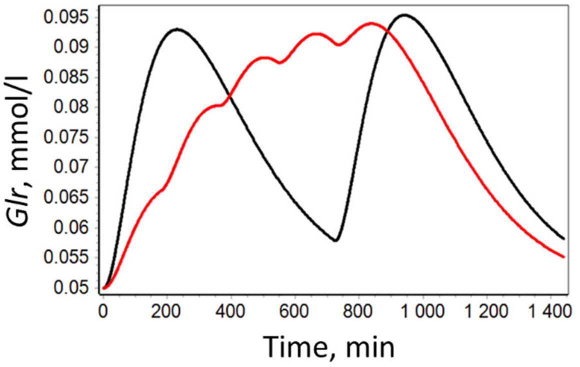
Glr, mmol/l
0.095
0.09
0.085
0.08
0.075
0.07
0.065
0.06
0.055
0.05
0 200 400 600 800 1 000 1 200 1 400
Time, min

(**v**)

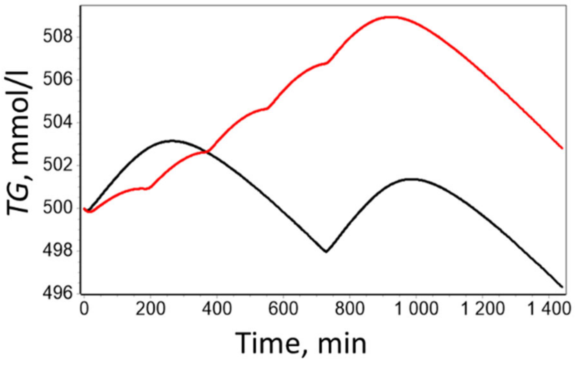
TG, mmol/l
508
506
504
502
500
498
496
0 200 400 600 800 1 000 1 200 1 400
Time, min

(**w**)

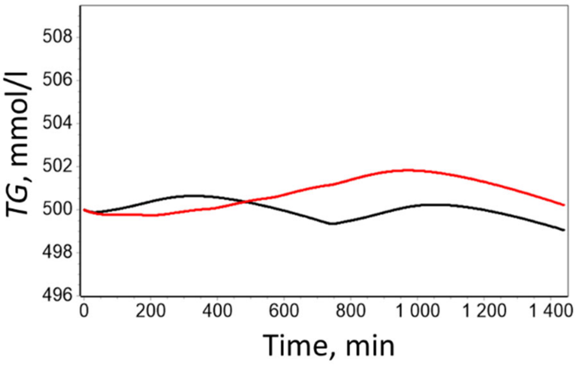
TG, mmol/l
508
506
504
502
500
498
496
0 200 400 600 800 1 000 1 200 1 400
Time, min

(**x**)

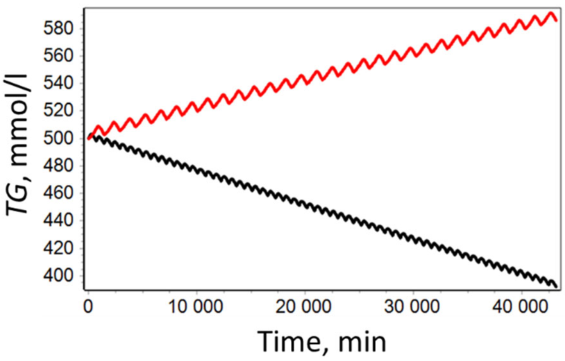

(**y**)

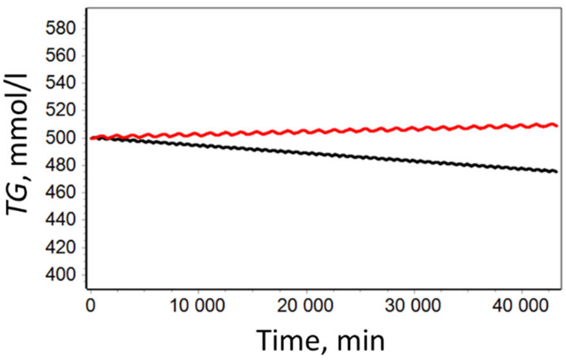

(**z**)

Figure S1. Dynamics of nutrients and metabolites in healthy individuals (left column) and patients with type 2 diabetes (right column) during two (black curves) and five (red curves) daily meals over 1440 min (one day) (**a**–**x**) and 43200 min (one month) (**y**, **z**) under identical daily caloric intake of 2047.7 kcal. Nutrients: (**a**, **b**) *C* – carbohydrates ingested with food; (**c**, **d**) *F* – fats ingested with food; (**e**, **f**) *Cs* – carbohydrates in the digestive system; (**g**, **h**) *Fs* – fats in the digestive system. Concentrations: (**i**, **j**) – glucose in plasma (*Gp*); (**k**, **l**) – insulin (*In*); (**m**, **n**) – triglycerides in plasma (*TGp*); (**o**, **p**) – fatty acids in plasma (*FAp*); (**q**, **r**) – glycerol-3-phosphate in adipocyte (*G3P*); (**s**, **t**) – fatty acids in adipocyte (*FA*); (**u**, **v**) – glycerol in adipocyte (*Glr*); (**w**, **x**) – triglycerides in adipocyte (*TG*).
